# Prostate cancer incidence and mortality among immigrants in Finland between 2000 and 2017 – a register-based cohort study

**DOI:** 10.2340/1651-226X.2025.43328

**Published:** 2025-06-29

**Authors:** Katja M. Mustonen, Maarit H. Lamminmäki, Tytti M. Sarkeala, Sirpa H. Heinävaara

**Affiliations:** a Faculty of Medicine, University of Helsinki, Helsinki, Finland; b Finnish Cancer Registry, Helsinki, Finland; c Faculty of Social Sciences, University of Tampere, Tampere, Finland; d Department of Public Health, Faculty of Medicine, University of Helsinki, Helsinki, Finland

**Keywords:** Prostate cancer, epidemiology, inequality, migrant health, registry data

## Abstract

**Background and purpose:**

Prostate cancer impacts millions of men worldwide each year, and its significance will continue to rise as populations age. Literature demonstrates differences in cancer burden between immigrant groups and non-immigrants across the world. Despite its prevalence, little research has focused primarily on prostate cancer among immigrants.

**Patients/material and methods:**

We utilized individual-level data on all immigrant men who had lived in Finland for over a year between 1973 and 2017 and aggregate data on Finnish-born men to determine immigrants’ incidence of and mortality from prostate cancer in relation to the men born in Finland. This gave us a study population of 162,844 non-Western and 56,127 Western immigrant men. Cases and deaths from the study period (2000–2017) were analyzed with the multivariate Poisson regression model for the groups, non-Western and Western immigrants separately.

**Results and Interpretation:**

Non-Western men had a relative risk (RR) of 0.663 (95% confidence interval [CI] 0.609–0.722) for cases and 0.803 (0.646–0.997) for deaths. Western men had RRs of 0.876 (0.784–0.978) and 0.78 (0.567–1.072), respectively. A longer duration of residence and a younger age at immigration increased the risk for prostate cancer. Compared to the men born in Finland, both immigrant groups showed a lower risk of prostate cancer. Non-Western men may have also had a lower risk of death from it. Prostate cancer mortality in non-Western immigrants appears to be high compared to its incidence. While uncertain, this implication is concerning enough to warrant further research into the topic.

## Introduction

Immigrants are often at a disadvantageous position in society [1], which has been linked to worse survival of many cancers [2]. In addition, although all who are residing in a municipality in Finland are entitled to highly affordable and comprehensive public health services [3], immigrants face unique barriers to accessing healthcare, including language barriers, cultural conflicts and misunderstandings, discrimination and prejudice, insufficient knowledge of available services, and worse quality of care [4–6]. Perhaps due to these barriers, immigrants utilize health services less than their locally-born counterparts [7]. Addressing these issues begins with providing policymakers and clinicians with accurate and specific information on the special characteristics of immigrants’ disease burden.

The relative size of the immigrant population of Finland increased by approximately 260% during our study period of 2000–2017, from around 2.7% of the total population to nearly 7.1% [8]. Most of these immigrants come from regions with different cultures and healthcare systems, as seen in Table 1. For these reasons, immigrant health is no longer a marginal issue, but warrants careful consideration.

**Table 1:** Person-years (pyrs) from each group and subgroup, rounded to the closest thousand, and corresponding percentages that the region contributes to its group.

Region of origin	Pyrs	Pyrs % of group
Native	33,958,000	100
Western		
Nordic	237,000	49.6
Other Western	241,000	50.4
Total	478,000	100
Non-Western		
Central and South Asia	102,000	8.2
East Asia and the Pacific	110,000	8.8
Latin America and the Caribbean	32,000	2.3
Middle East and North Africa	215,000	17.2
Sub-Saharan Africa	139,000	11.2
Eastern Europe	648,000	52.1
Total	1,390,000	100

According to estimates, there are 1.4 million new prostate cancer (PCa) cases and 375,000 deaths annually worldwide [9]. PCa is the second most common non-skin malignancy in the world, and the fifth most common cause of cancer death in men [9]. However, incidence and mortality rates exhibit significant variation between regions: both are generally highest in Western countries, with the exception of many sub-Saharan African regions and the Caribbean, which in addition to a high incidence, exhibit exceptionally high mortalities [9]. An important cause for the differences in incidence is the abundant and controversial use of prostate-specific antigen (PSA) testing [10], which varies starkly between countries [9]. Very few countries, Finland not among them, have ever implemented organized PCa screening programs, and debate over its advantages and disadvantages is ongoing [11].

Due to PCa’s strong association with advanced age [12], its impact on disability-adjusted life years will grow in the future as different populations age and life-expectancies grow [13], underlining the importance of understanding all aspects of the disease. Previous studies from around the world have examined different aspects of immigrant health extensively [14–16], including many cancer-focused studies [17, 18]. However to our knowledge, none have studied PCa among immigrants in Finland, even though there have been results from other Nordic countries of differences between non-immigrants and immigrants [17].

The aim of our study is to establish immigrants’ relative risk (RR) of and death from PCa in Finland in comparison to that of men born in Finland. To this end, we compared the PCa risks of Western and non-Western immigrants separately to those of the men born in Finland using long-term registry data analyzed with the Poisson regression model.

## Materials and methods

We compared the incidence of and mortality from prostate cancer (ICD code C61) among immigrant men in Finland between the years 2000 and 2017 to that of Finnish men. We first gathered individual-level data on immigrant men from the Finnish Digital and Population Data Services Agency (DPDSA) and Statistics Finland. Individual data on all men with PCa were gathered from Finnish Cancer Registry (FCR) databases.

**Figure 1:**
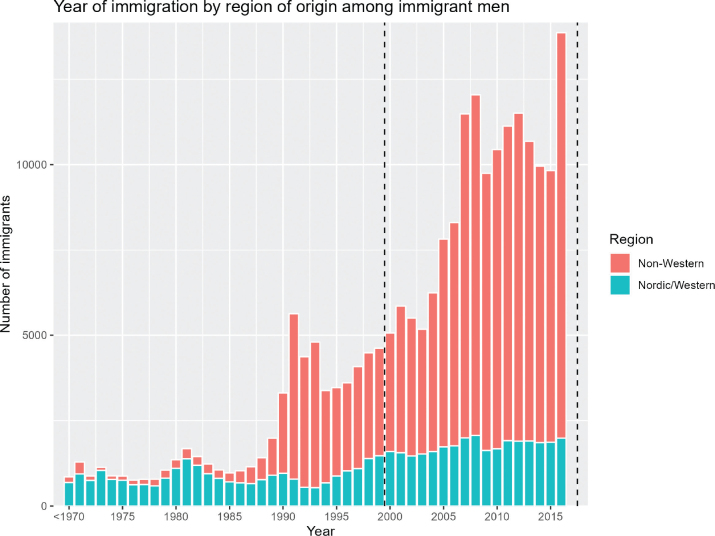
Number of male immigrants by year of immigration. Dotted lines show the follow-up period.

An immigrant was defined as an individual born outside Finland. From the DPDSA, we extracted data on the birth country of all immigrant men who had lived in Finland between 1973 and 2017 (Figure 1). These data were linked with first primary PCa diagnoses and deaths obtained for the same period. The data on the causes of death were obtained from Statistics Finland. The data on possible emigrations of immigrants were obtained from the DPDSA.

Immigrants were divided into the groups, non-Western and Western. The non-Western group was pooled from more specific subgroups of Central and South Asia, East Asia and Pacific, Latin America and Caribbean, Middle East and North Africa, Eastern Europe, and sub-Saharan Africa. The Western group came from the subgroups: Nordic and Western. A complete list of which countries of origin of the immigrant men were classified into which subgroups for analysis can be found in the Supplementary Material.

For immigrants, we aggregated individual data on cancer diagnoses, cancer deaths, and follow-up time by 10-year age group, 10-year calendar period, and region of birth. The data on PCa diagnoses and deaths of the men born in Finland were aggregated by the same age groups and calendar periods.

We did not have individual level follow-up data on men born in Finland, and thus we approximated their person-years (pyrs) by subtracting immigrants’ pyrs from the annual mean population counts for each year of the study period obtained from Statistics Finland.

**Figure 2:**
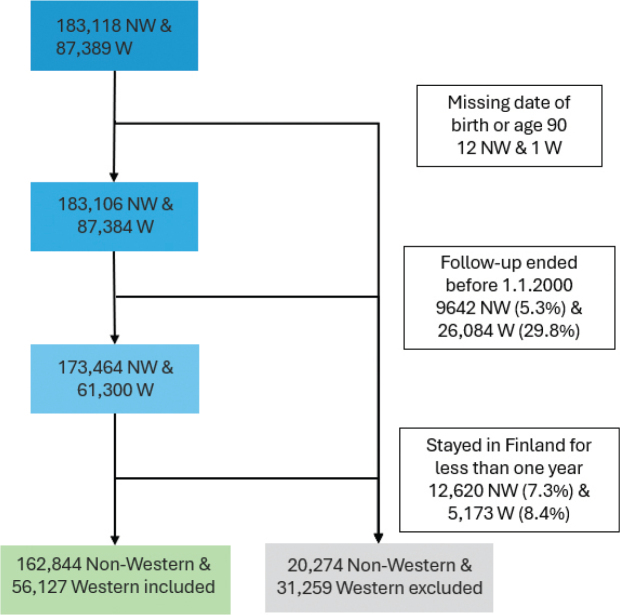
The formulation of the study population of non-Western (NW) and Western (W) immigrants in Finland in 2000–2017. Individuals were excluded from the study population on the following grounds: 1. Missing date of birth, 2. age 90 or older, 3. follow-up ended before 1.1.2000, and 4. stayed in Finland for less than a year. Percentages are shown for those >1%.

An immigrant was excluded from the data if his date of birth was missing, he was aged more than 90, his follow-up ended before 2000, or he had stayed in Finland for less than a year (Figure 2). Thus, out of 183,118 non-Western immigrants, 162,844, and out of 87,389 Western immigrants, 56,127 were included after these exclusions. In total, 20,274 (11.1%) and 31,262 (35.8%) were excluded, respectively.

If the individual had immigrated to Finland before the year 2000, his follow-up began on January 1^st^, 2000. Otherwise, his follow-up began at immigration. An individual’s follow-up was ended at death, emigration from Finland, or age 90, whichever occurred first. For the incidence analysis, follow-up ended also at the time of PCa diagnosis.

We used a multivariate Poisson regression model to compare 10-year age- and calendar-specific incidence and mortality between the immigrants and the men born in Finland. These analyses were adjusted for the region of origin, age group and calendar period. These calculations were also done separately for both the Western and the non-Western groups. In addition, we also studied the trend between the categories. All data processing and analyses were performed using the R program version 4.3.2.

We report the results by RRs with their 95% confidence intervals (CI) (shown in parenthesis). Among immigrants, we calculated how the RR of being diagnosed and dying of PCa changed by the duration of residence and the immigration age. To do this, we grouped the cases and the deaths into the categories 1–9, 10–19, and 20+ years for the duration of residence, and into 1–19, 20–29, 30–39, and 40+ years for the immigration age.

## Results

In our study population of 56,127 Western and 162,844 non-Western immigrant men, there were roughly 0.5 million pyrs in the Western and 1.4 million pyrs in the non-Western group. In terms of pyrs, the Western group was divided into equally sized subgroups, Nordic and other Western. However, in the non-Western group, the subgroup Eastern Europe dominated over the five other subgroups, and Latin America and Caribbean stood out as insignificant to the whole. The other subgroups were comparatively equal in sizes (Table 1). Baseline descriptives of the study populations are provided in Table 2.

**Table 2:** Baseline descriptives of the study population of immigrants

Baseline description the study population of immigrants	Western		Non-Western	
	*N*	%	*N*	%
**Birth cohort**				
-1949	3,732	6.6	6,536	4.0
1950–1959	3,642	6.5	10,742	6.6
1960–1969	8,025	14.3	22,512	13.8
1970–1979	14,951	26.6	34,488	21.2
1980–1989	12,524	22.3	47,023	28.9
1990–1999	6,130	10.9	24,287	14.9
2000–2009	5,207	9.3	13,274	8.2
2010–2017	1,916	3.4	3,982	2.4
**Age at immigration**				
0–9	22,750	40.1	25,005	15.4
10–19	4,233	7.5	23,722	14.6
20–29	14,305	25.5	57,333	35.2
30–39	8,982	16.0	34,619	21.3
40–49	3,493	7.0	14,393	8.8
50–59	1,441	2.6	5,219	3.2
60–69	728	1.3	1,822	1.1
70–79	163	0.3	578	0.4
80–89	32	< 0.1	81	< 0.1
**Duration of residence**				
0–9	24,652	43.9%	92,704	56.9%
10–19	11,148	19.9%	43,864	26.9%
≥ 20	20,327	36.2%	26,276	16.1%

Among men born in Finland, there were 81,363 PCa cases and 13,484 deaths. In the non-Western immigrant group, there were 536 cases and 82 deaths, and in the Western immigrants, 318 cases and 38 deaths (Table 3).

**Table 3:** Person-years at risk (pyrs), number of observations, relative risk (RR) and corresponding 95% confidence intervals (CI) of cases of and deaths from PCa of immigrant groups and subgroups. RRs are adjusted for the region of origin, age group and calendar period. Figures are marked with a dash if there were fewer than five observations.

Region of origin	Pyrs	*N* cases	RR of cases	CI of cases	*N* deaths	RR of deaths	CI of deaths
Native	33,958,000	81,363	1	-	13,484	1	-
Western	478,000	318	0.88	0.78–1.00	38	0.78	0.57–1.11
Nordic	237,000	125	1.02	0.85–1.21	12	0.81	0.46–1.43
Other Western	241,000	193	0.80	0.70–0.93	26	0.77	0.52–1.13
Non-Western	1,390,000	536	0.66	0.61–0.72	82	0.80	0.65–1.00
Central and South Asia	102,000	10	0.31	0.17–0.58	-	-	-
East Asia and the Pacific	110,000	17	0.32	0.20–0.52	-	-	-
Latin America and the Caribbean	32,000	14	0.93	0.55–1.58	-	-	-
Middle East and North Africa	215,000	42	0.45	0.33–0.61	5	0.595	0.248–1.43
Eastern Europe	648,000	414	0.72	0.66–0.80	71	0.89	0.71–1.13
Sub-Saharan Africa	139,000	39	0.91	0.66–1.24	-	-	-

Compared to the men born in Finland, the incidence of PCa was significantly lower in non-Western men: an adjusted RR of 0.66 (0.61–0.72) was observed. The RR of the mortality from PCa was 0.80 (0.65–1.00) (Table 3). In the Western group, the incidence was also lower than in the men born in Finland (0.88, 0.78–0.98). However, the mortality RR in Western men, based on a rather small number of observations, was statistically insignificant (0.78, 0.57–1.11).

In the non-Western group, a majority of both cases (77.2%) and deaths (86.6%) came from the subgroup Eastern Europe. Conversely, RRs for cases were strikingly low across all Asian subgroups. There were next to no deaths in subgroups other than Eastern Europe. A complete table of the absolute figures for all subgroups can be found in Supplementary Table 1.

We also found that for non-Western immigrants, the likelihood of being diagnosed with PCa was greatest if the duration of residence was 20 years or longer (Table 4). Additionally, we found that the risk of being diagnosed with PCa was inversely proportional to age at immigration for non-Western immigrants: the younger the immigrant was at immigration, the greater his risk of PCa. Neither of these findings were seen in the Western immigrant group. Similar figures of deaths cannot be reported due to the small number of them.

**Table 4:** A. Relative risk (RR) of cases by duration of residence and corresponding 95% confidence intervals (CI). B. RR of cases by age at immigration and corresponding CI. Trends in both A. and B. are adjusted by calendar period, age group, and region. These are linear trends by category medians.

A. Duration of residence	*N* cases	RR	CI
***Non-Western***			
0–9 (ref.)	155	1	N/A
10–19	164	1.00	0.80–1.25
20+	217	1.36	1.09–1.68
Trend	-	1.17	1.05–1.31
***Western***			
0–9 (ref.)	55	1	N/A
10–19	45	1.04	0.70–1.54
20+	218	1.07	0.79–1.44
Trend	-	1.03	0.90–1.19
***All immigrants***			
0–9 (ref.)	210	1	N/A
10–19	209	1.00	0.83–1.21
20+	435	1.24	1.04–1.5
Trend		1.12	1.03–1.22
B. Age at immigration	*N* cases	RR	CI
***Non-Western***			
0–19	< 5	-	-
20–29	41	1.49	1.05–2.11
30–39	120	1.31	1.05–1.63
40+ (ref.)	374	1	N/A
Trend	-	0.81	0.70–0.94
***Western***			
0–19	10	0.55	0.27–1.12
20–29	73	1.22	0.89–1.67
30–39	89	1.04	0.79–1.37
40+ (ref.)	146	1	N/A
Trend	-	0.10	0.87–1.15
***All immigrants***			
0–19	11	0.68	0.36–1.28
20–29	114	1.39	1.10–1.74
30–39	209	1.18	0.99–1.40
40+ (ref.)	520	1	N/A
Trend	-	0.90	0.81–1.00

## Discussion

Our study sought to find possible differences in the relative incidence of and mortality from PCa between men born in Finland and immigrant men. We found that the incidence of PCa is lower in non-Western immigrants than in the men born in Finland. For the non-Western immigrants, we found a lower incidence compared to men born in Finland. Our result for the mortality from PCa seems to also be lower than that of the men born in Finland. However, caution must be used in interpreting this finding, as the CI is broad (RR = 0.80, CI = 0.65–1.00). In Western immigrants, the incidence of PCa was lower than the men born in Finland, but no statistically significant difference was observed for PCa mortality in this group.

PCa often remains latent without causing a clinically significant disease during the patient’s lifetime [19]. Because of widespread opportunistic PSA testing, overdiagnosis is common and causes undue anxiety for patients [20]. Testing varies greatly due to differences in accessibility of healthcare and even clinical practices [10, 21], which can be seen in PCa incidences between different countries. For example, Estonia’s high PCa incidence is largely explainable by the largescale opportunistic PSA testing employed by clinicians [21].

Northern European countries generally have high incidence rates, and Finland is no exception [22]. This is due to either increased risk of PCa, pervasive overdiagnosis, or both. Overdiagnosis among men born in Finland may help to explain the differences we observed: diagnoses of clinically insignificant cancers would inflate the perceived incidence of PCa. It can be hypothesized that immigrants might be subject to this less due to a lower utilization of healthcare [7], leading to fewer diagnosed PCa cases.

We found that Western immigrants had a higher incidence than non-Westerners, which is congruent with other studies’ findings [17, 23]. Literature shows that cultural similarities between the host population and immigrant groups may predict less inequality in healthcare [24]. Thus, it is reasonable to theorize that if overdiagnosis of PCa is associated with the utilization of healthcare, it could in part explain this observation. Still, other factors must be considered as well [25].

For a disease as common as PCa, surprisingly little is known of its risk factors. Advanced age and a family history are known to be important non-modifiable risk factors [12]. Many modifiable risk factors have been proposed, but only a handful have been widely accepted as certain. Often cited ones include meat consumption, dietary inflammation index, and trans fatty acid intake. Plentiful soy consumption and exercise are known to be protective factors [26, 27]. Indeed, the Western lifestyle has been linked to increased risk of prostate cancer, which may in part explain the lower incidence in non-Western immigrants compared to both the men born in Finland and Western immigrants seen in our study.

Our results show that non-Western immigrants had a lower risk of diagnosis than the men born in Finland. This may be explained by a worse socioeconomic position [1], lacking knowledge of available services [6], and barriers in communication between healthcare professionals and patients [4].

Our finding of a positive association between long duration of residence and young age at immigration and higher RR of diagnosis in non-Western men is consistent with a Swedish study [28]. The same study also demonstrates the effect of integration into society on PCa risk in immigrants. Another Swedish paper concludes that a young age at immigration predicts better social integration [29]. Therefore, it can be interpreted that young immigrants adjust well to the culture and lifestyle of the host country, which results in epidemiological findings similar to the Finnish-born population.

It can be assumed that immigrants who live in Finland longer are exposed to environmental and lifestyle-related risk factors for longer. The fact that we did not see similar effects in the Western immigrants’ RRs supports the theory that cultural differences are less of an obstacle for them, and that they are exposed to the same risk factors associated with the Western lifestyle as the men born in Finland in our study even before immigration. These findings on the effect of age at immigration and duration of residence are supported by literature [23].

Our findings are congruent with the literature in demonstrating a lower PCa risk in non-Western immigrants [30]. However, non-Western immigrants are a highly heterogenous group which includes people from all over the world and from many different backgrounds. This diversity is well demonstrated also in our results, with Asians standing out with very low RRs for cases, and conversely Eastern Europeans and sub-Saharan Africans having an above average RR for cases among the non-Western subgroups. These results are to be expected based on epidemiological [9] and genetic studies [25].

In our study, the majority of the non-Western cases and deaths came from the subgroup Eastern European. This overrepresentation is partially explained by the small study populations in other non-Western subgroups, but it also has basis in literature: the finding of an above average risk of PCa in Eastern Europeans compared to other non-Western immigrants is consistent with a Norwegian study [17].

Concerningly, another Norwegian study also found a worse survival of PCa among Eastern European immigrants [31], while other studies have found ambiguous results in the incidence of PCa [30]. Our study does not unambiguously demonstrate a worse PCa survival in immigrants, but it is possible in non-Western immigrants, and it is clear that these immigrants do not survive their cancers better than the men born in Finland. Further research should be conducted into this matter in the future.

A major strength of our study is the quality of our data. The register-data we utilized are reliable, and the data from the FCR are comprehensive even with its follow-ups [32]. However, we were not able to assess the potential effect of opportunistic PSA testing via the stage of PCa since the stage classification adapted by the FCR is unfortunately too crude for that purpose.

Our study was limited by the number of observations of both immigrant PCa cases and deaths, due to the low number of immigrants in Finland and the relative youth of this population. While the stark differences in the age distributions of men born in Finland [33] and immigrant men did not affect our results due to adjustment for age group, the low amount of elderly immigrants decreased the number of observations in these groups, since PCa is typically diagnosed late in life [34]. Individual-level data on Finnish men were unavailable to us and as such we utilized aggregate data. This may have affected our data to a small degree, but this is likely a marginal issue and does not impact our results.

We were unable to reliably determine the effect of duration of residence and age at immigration on the mortality from PCa, mainly due to the small number of deaths on record. Additionally, in the case of age at immigration, meaningful analysis was impossible because nearly all of the deaths occur in the oldest age group. This is likely because the men who immigrated at a younger age have not yet reached an age at which PCa mortality is observed, as few immigrated into Finland before the year 1990 (Figure 1).

Encouragingly, our results indicate that immigrants are at a lower risk of both incidence of and mortality from PCa compared to the men born in Finland. However, our findings do not rule out a worse PCa survival among non-Western immigrants, especially Eastern European ones which has been observed in Norway [31]. This may be due to factors such as immigrants’ cancers being detected at a later stage [35]. Further research is needed into this possibility, for example by comparing the survival from similar grade cancers between immigrants and men born in Finland.

## Supplementary Material



## Data Availability

Aggregated data are available from the corresponding author upon reasonable request. Due to data protection regulations, the register data are not openly shared. Permission for the dataset can be applied for from Findata (https://findata.fi/en/permits/).
