# Supplementary material for: Prostate cancer incidence and mortality among immigrants in Finland between 2000 and 2017 – a register-based cohort study
Source: Acta Oncol. 2025 Jun 29;64:43328. doi: 10.2340/1651-226X.2025.43328 (PMC12228077; doi:10.2340/1651-226X.2025.43328)
Supplement: Supplementary file 1 [file AO-64-43328-s1.pdf]

Supplementary material has been published as submitted. It has not been copyedited, or typeset by Acta Oncologica

Region of origin	N individuals	N cases	N deaths
**Native**	**-**	**81,363**	**13,484**
Nordic	21,086	125	12
Other Western	35,041	193	26
**Total Western**	**56,127**	**318**	**38**
Central and South Asia	16,397	10	<5
East Asia and Pacific	*16,495*	17	<5
Latin America and Caribbean	4,501	14	<5
Middle East and North Africa	27,835	42	5
Sub-Saharan Africa	18,553	39	<5
Eastern Europe	79,064	414	71
**Total Non-Western**	**162,844**	**536**	**82**

Appendix table 1. Numbers (N) of individuals, cases, and deaths in each group and subgroup.

Group	Countries and Territories
**Western**	
Nordic	Denmark, Iceland, Norway, Sweden
Other Western	Andorra, Australia, Austria, Belgium, Canada, Cyprus, France, Germany, Greece, Ireland, Italy, Liechtenstein, Luxembourg, Malta, Monaco, Netherlands, New Zealand, Portugal, San Marino, Spain, Switzerland, United Kingdom, United States of America, Vatican City
**Non-Western**	
Central and South Asia	Afghanistan, Armenia, Azerbaijan, Bangladesh, Bhutan, Ceylon, Georgia, India, Kazakhstan, Kyrgyz Republic, Maldives, Nepal, Sri Lanka, Tajikistan, Turkmenistan, Uzbekistan
East Asia and Pacific	American Samoa, Brunei, Cambodia, China, Fiji, Hong Kong, Indonesia, Japan, Korea Dem. People's Rep., Lao PDR, Macao, Malaysia, Mongolia, Myanmar, Papua New Guinea, Philippines, Republic of Korea, Samoa, Singapore, Solomon Islands, South Vietnam, Taiwan, Thailand, Vietnam
Latin America and Caribbean	Antigua and Barbuda, Antilles, Argentina, Aruba, Bahamas, Barbados, Belize, Bolivia, Brazil, Chile, Colombia, Costa Rica, Cuba, Curacao, Dominican Republic, Ecuador, El Salvador, Grenada, Guatemala, Guyana, Haiti, Honduras, Jamaica, Mexico, Nicaragua, Panama, Paraguay, Peru, Puerto Rico, St. Kitts and Nevis, St. Lucia, St. Martin, Suriname, Trinidad and Tobago, Turks and Caicos Islands, Uruguay, Venezuela

Middle East and North Africa	Algeria, Bahrain, Egypt, Iran, Iraq, Israel, Jordan, Kuwait, Lebanon, Libya, Morocco, Oman, Palestine, Qatar, Saudi Arabia, South Yemen, Syria, Tunisia, Turkey, United Arab Emirates, Yemen
Eastern Europe	Albania, Belarus, Bosnia and Herzegovina, Bulgaria, Croatia, Czech Republic, Czechoslovakia, Estonia, Hungary, Kosovo, Latvia, Lithuania, Moldova, Montenegro, North Macedonia, Poland, Romania, Russia, Serbia, Slovak Republic, Slovenia, Soviet Union, Ukraine, Yugoslavia
Sub-Saharan Africa	Angola, Benin, Botswana, Burkina Faso, Burundi, Cabo Verde, Cameroon, Central African Republic, Chad, Comoros, Congo Republic, Cote d'Ivoire, Djibouti, Equatorial Guinea, Eritrea, Eswatini, Ethiopia, Gabon, Gambia, Ghana, Guinea, Guinea-Bissau, Kenya, Liberia, Madagascar, Malawi, Mali, Mauritania, Mauritius, Mozambique, Namibia, Niger, Nigeria, Rhodesia, Rwanda, Senegal, Seychelles, Sierra Leone, Somalia, South Africa, South Sudan, South West Africa, Sudan, Tanzania, Togo, Uganda, Zaire, Zambia, Zimbabwe

Appendix Table 2. Groupings of countries and territories
